# *CDKL5* gene status in female patients with epilepsy and Rett-like features: two new mutations in the catalytic domain

**DOI:** 10.1186/1471-2350-13-68

**Published:** 2012-08-06

**Authors:** Hiart Maortua, Cristina Martínez-Bouzas, María-Teresa Calvo, Maria-Rosario Domingo, Feliciano Ramos, Ainhoa García-Ribes, María-Jesús Martínez, María-Asunción López-Aríztegui, Nerea Puente, Izaskun Rubio, María-Isabel Tejada

**Affiliations:** 1 Laboratorio de Genética Molecular, Servicio de Genética, Hospital Universitario Cruces, Instituto BioCruces, Barakaldo-Bizkaia, Spain; 2 Unidad de Genética Médica, Hospital Universitario Miguel Servet, Zaragoza, Spain; 3 Sección de Neuropediatría del Servicio de Pediatría, Hospital Universitario Virgen de la Arrixaca, Murcia, Spain; 4 Consulta de Genética Clínica, Departamento de Pediatría, Hospital Clínico de Zaragoza, Zaragoza, Spain; 5 Sección de Neuropediatría del Servicio de Pediatría, Hospital de Cruces, Barakaldo-Bizkaia, Spain; 6 Laboratorio de Citogenética y Consulta de Consejo Genético, Servicio de Genética, Hospital Universitario Cruces, Barakaldo-Bizkaia, Spain

**Keywords:** *CDKL5*, Epilepsy, *MECP2*, MLPA, Rett syndrome

## Abstract

**Background:**

Mutations in the cyclin-dependent kinase-like 5 gene (*CDKL5*) located in the Xp22 region have been shown to cause a subset of atypical Rett syndrome with infantile spasms or early seizures starting in the first postnatal months.

**Methods:**

We performed mutation screening of *CDKL5* in 60 female patients who had been identified as negative for the methyl CpG-binding protein 2 gene (*MECP2*) mutations, but who had current or past epilepsy, regardless of the age of onset, type, and severity. All the exons in the *CDKL5* gene and their neighbouring sequences were examined, and *CDKL5* rearrangements were studied by multiplex ligation-dependent probe amplification (MLPA).

**Results:**

Six previously unidentified DNA changes were detected, two of which were disease-causing mutations in the catalytic domain: a frameshift mutation (c.509_510insGT; p.Glu170GlyfsX36) and a complete deletion of exon 10. Both were found in patients with seizures that started in the first month of life.

**Conclusions:**

This study demonstrated the importance of *CDKL5* mutations as etiological factors in neurodevelopmental disorders, and indicated that a thorough analysis of the *CDKL5* gene sequence and its rearrangements should be considered in females with Rett syndrome-like phenotypes, severe encephalopathy and epilepsy with onset before 5 months of age. This study also confirmed the usefulness of MLPA as a diagnostic screening method for use in clinical practice.

## Background

Rett Syndrome (RTT) is a neurodevelopmental disorder characterized by loss of spoken language and hand use, hand stereotypes, and mental retardation [1], and is the second most common genetic cause of severe mental retardation in females [2]. Although mutations in the methyl CpG-binding protein 2 gene (*MECP2*) can be found in 95–97% of individuals with classical RTT and in 50–70% of those with atypical RTT [1], some patients do not carry mutations in this gene, suggesting the existence of other genetic causes of RTT [3].

The cyclin-dependent kinase-like 5 gene (*CDKL5*) is located in the Xp22 region and has been found to be associated with atypical RTT with infantile spasms or early seizures starting in the first postnatal months (Hanefeld variant) [4,5]. *CDKL5* is composed of 20 coding exons and codes for a protein of 1,030 amino acids [6]. *CDKL5* mRNA is highly expressed in the adult human brain, which is indicative of its importance in neuronal function and development [3].

The clinical overlap between patients with mutations in *CDKL5* and patients with RTT caused by mutations in *MECP2* reflects the fact that these genes belong to the same pathway [7,8]. To date, more than 80 cases of pathogenic *CDKL5* mutations have been reported [9], but it is likely that many more exist [7]. The present study investigated mutations in *CDKL5* in a cohort of patients with epilepsy and RTT or other RTT-like phenotypes to improve the diagnostic criteria for these patients and to clarify the pathological mechanisms of *CDKL5* mutations.

## Methods

### Patients

Patients were referred to our diagnostic laboratory for investigation of *MECP2* gene status. We performed mutation screening of *CDKL5* in 60 females from this group who were negative for mutations and large rearrangements in *MECP2*, and who had current or previous epilepsy, regardless of the age of onset, type, or severity. Among these, eight patients were clinically diagnosed with classic RTT, and one with atypical RTT. Twenty patients were thought to be Angelman-syndrome but they were negative for mutations and deletions in *UBE3A*, and six presented with autistic features. The spectrum of phenotypes of the remaining patients was heterogeneous but with Rett-like features.

These patients were referred by various paediatric neurologists and clinical geneticists throughout Spain, and were included in the present study after signed informed consent forms were obtained from the patients’ parents.

One hundred DNA samples from anonymous healthy female individuals were used as normal controls for exons 1, 12, 17 and 21 (200 X chromosomes). The samples were obtained from the Basque Centre for Transfusions and Human Tissues. The samples and associated data were processed and released by the Basque Biobank for Research-OEHUN (http://www.biobanco.org) following standard operating procedures with appropriate ethical approval. The control individuals had similar geographic origins to the patients. The whole project was approved by the Ethics Committee of Cruces University Hospital.

### Screening for *CDKL5* mutations

*CDKL5* coding sequences and each intron/exon boundary were amplified by polymerase chain reaction (PCR), using previously described primers [10-12], with slight modifications of the amplification conditions. The PCR products were analysed using conformation-sensitive gel electrophoresis (CSGE), after silver-staining following our protocols [13]. Sequence analysis of genomic fragments with CSGE mobility shifts was carried out on an ABI PRISM 3130xl automated DNA sequencer (Applied Biosystems). Exons 4 and 6 were sequenced directly.

### Rearrangements studies

Genomic rearrangements were examined using multiplex ligation-dependent probe amplification (MLPA) kits (P189-A2 and P189-B1, MRC Holland, The Netherlands), according to the manufacturer’s protocols.

### RNA isolation and reverse-transcriptase PCR

We used cDNA to study mutations/variations in exons 8 and 12. Total RNA isolation and cDNA synthesis were performed as described previously [13]. The region from exon 8 to exon 12 of *CDKL5* was amplified by PCR using the following flanking primers: forward, 5′-CAGAGTACGTTGCCACCAGA-3′ in exon 8; and reverse, 5′-GCAGGCCTACACTCAGGTTC-3′ in exon 12. The primers used to amplify only exon 12 were: forward, 5′-TGCACACCAAAACCTACCAAGC-3′ at the start of exon 12; and reverse, 5′-GAATGGCTACTGTCCATGTGC-3′ at the end of exon 12.

In both cases, the PCR products were directly sequenced on both strands using a BigDye Terminator Kit (Applied Biosystems) in a 3130xl automated sequencer (Applied Biosystems).

### X chromosome inactivation

X-chromosome inactivation was studied by examination of the highly polymorphic small tandem repeat within the human androgen receptor gene, using previously described protocols [14].

## Results

The pathogenic mutations, variants, and polymorphisms identified in the current study are listed in Table 1. Six of them represent DNA changes not previously described in any of the 10 databases consulted [15-24]. Two of these new changes were pathogenic, disease-causing mutations found in two patients with early-onset epileptic seizures within the first month of life, and were both *de novo* mutations not present in their parents.

**Table 1:** CDKL5 mutation/variant identified in this study

Total number of females studied*	Number of DNA variants*	**Location**	**Nucleotide change**	**Aminoacid change**	**Domain**	**Effect**	Reference**
60 p	1 p	Exon 8	c.509_510insGT	p.Glu170GlyfsX36	Catalytic	Pathogenic	NEW, this study
60 p	1 p	Exon 10	c.745-?_825 + ?del	----------	Catalytic	Pathogenic	NEW, this study
160 (60p + 100c)	1 p	Exon 12	c.1455_1460delGGCCAA	p.Ala486_Lys487del	C-Ter	Unknown Variation	NEW, this study
160 (60p + 100c)	7 (1p + 6 c)	Exon 17	c.2389 G > A	p.Asp797Asn	C-Ter	Polymorphism	NEW, this study
160 (60p + 100c)	6 (1p + 5 c)	Before exon 1	c.-426 C > G	----------	------	Polymorphism	NEW, this study
60 p	1 p	Intron 6	c.403 + 27A > G	----------	------	Without pathogenic effect	NEW, this study
160 (60p + 100c)	2 (1p + 1c)	Exon 21	c.2995 G > A	p.Val999Met	C-Ter	Polymorphism (SNP: rs35693326)	Nectoux et al. [26] Intusoma et al. [27]
160 (60p + 100c)	6 (2p + 4c)	Before exon 1	c.-391 G > T	----------	------	Polymorphism	Evans et al. [12]
60 p	2 p	Exon 4, 21	c.145 + 17A > G; c. 3003 C > G; c. 3084 G > A	p.His1001His; p.Thr1028Thr	Catalytic, C-Ter	Polymorphism	Tao et al. [28]

*p: Female patients; c: control healthy females.

** New mutations/variants have not been described in any of the 10 Genome databases revised [15-24].

The first of these two new mutations was a pathogenic frameshift mutation in exon 8 (c.509_510insGT; p.Glu170GlyfsX36) producing a truncating protein in the catalytic domain that contains only 206 amino-acids on the 1,030 that has CDKL5 protein. The patient was the first child of healthy parents. She was born in a private clinic, and we were unable to obtain all the details related to the delivery, but established that resuscitation had not been required and the infant’s birth weight was 3,450 g. The infant started to have seizures at 4 weeks of life, with facial flushing, vomiting and crying. The family contacted us when the patient was 11 years old with a clinical diagnosis of RTT, with severe mental and psychomotor retardation, poor social contact, axial hypotonia, spastic tetraparesis and epilepsy refractory to numerous treatments.

**Figure 1:**
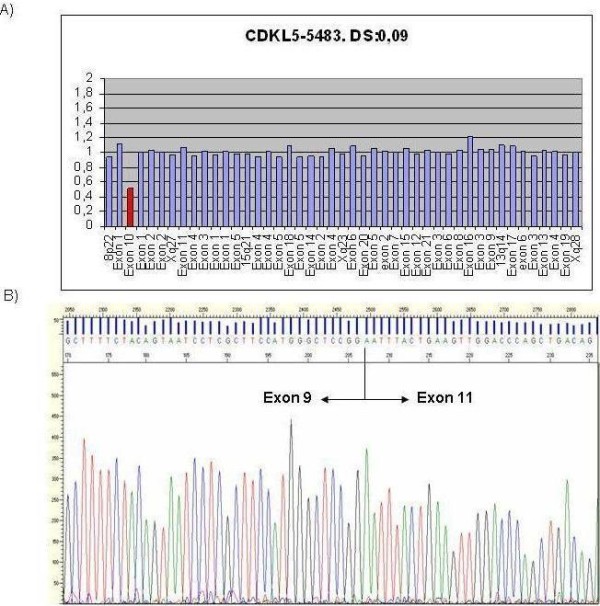
Complete deletion of exon 10 detected by MLPA (A) and confirmed by cDNA analysis (B).

The second new mutation was a complete deletion of exon 10 (p.745-?_825 + ?del) detected by MLPA (Figure 1A) and confirmed by cDNA analysis (Figure 1B). Although this was an in-frame deletion, we considered it likely to be pathogenic for several reasons: 1) it was a *de novo* mutation; 2) all exon deletions from exons 1 to 15 in the genomic databases consulted [16,17,19-21] were pathogenic; 3) it was located within the catalytic domain; and 4) the clinical description of the patient was consistent with that of others with *CDKL5* deletions. This patient was referred to the Clinical Genetics Unit at 4 years old because of delayed psychomotor development, gait abnormalities, growth retardation and severe mental retardation. She was the first child of healthy parents, born by normal delivery after an uncomplicated pregnancy. She had her first epileptic seizures at 4 weeks old (2–3 times a day) and these seizures were refractory to treatment. Follow-up until the most recent visit, at age 6 years, indicated severe and progressive deterioration of her psychomotor development.

Four other new DNA variants were identified, including c.1455_1460delGGCCAA (loss of lysine and alanine amino acids) in exon 12. This patient was referred to us at 3 years 2 months old by a paediatric neurologist because of developmental delay, absence of speech, seizures, and difficulty in walking (possible ataxia), though her initial psychomotor development was normal. She was the second child of healthy parents, and had a healthy 13-year-old sister. She had her first febrile seizure at the age of 11 months, and her mother reported that her development “stopped” after this episode, including with respect to her speech (possible regression). We initially classified this DNA variant as non-pathogenic because her asymptomatic mother had the same variant, and both the mother and infant showed the same cDNA and random X chromosome inactivation. However, this variant was not present in the 10 genomic databases consulted, or in the 100 normal control samples, and we therefore reclassified it as an unknown variation.

The second DNA variant was c.2389 G > A (p. Asp797Asn) in exon 17. This was inherited from the father and was therefore not a pathogenic mutation. Six other females in the healthy control group also showed this change, indicating that it represents a newly-described polymorphism in Spain.

The remaining two novel DNA changes had no clinical significance: c.−426 C > G and c.403 + 27A > G. The first was also found in five healthy female controls, and thus represented another novel polymorphism for Spain. *In silico* studies of the second change using the ESE Finder program [25] indicated that it was non-pathogenic. Furthermore, a similar change (c.403 + 80 G > A) in the 1,000 genomes database [24] was classified as a single nucleotide polymorphism (SNP) variant. No parental DNA was available for either of these cases.

Finally, we detected three previously reported variants. The variant c.2995 G > A (p.Val999Met), first described by Nectoux *et al.*[26] as likely non-pathogenic and later classified as a polymorphism [27] and SNP (rs35693326 [18]), was found in a patient whose asymptomatic mother also had this variant. This variant was also identified among the control group, and we therefore concluded that it represented a gene polymorphism. c.−391 G > T first reported by Evans *et al.*.[12] was found in two patients and four controls. Finally, the haplotype c.145 + 17A > G; c. 3003 C > G and c. 3084 G > A, first described by Tao J *et al.*[28], was found in another two patients.

## Discussion

Kalscheuer *et al.*[11] in 2003 provided the first report of mutations in the *CDKL5* gene in two unrelated patients with infantile spasms and mental retardation due to two different balanced X-autosome translocations; since then, more than 80 patients with pathogenic mutations in this gene have been described [29]. The current study analysed *CDKL5* mutations in 60 female patients with epilepsy, initially thought to be associated with *MECP2* mutations, but who were negative for mutations and large rearrangements in this gene.

This complete genetic study of *CDKL5* (sequencing of all the exons and their neighbouring sequences and analysis of gene rearrangements by MLPA) identified two new, previously undescribed pathogenic mutations, consisting of one frameshift mutation and one deletion (2/60, 3%). This rate is lower than those reported by some other authors (7,6–8%) [7,8,30], but these previous studies only included patients with precocious epilepsy, while the current study included patients undergoing *MECP2* screening, but with any type of epilepsy regardless of the age of onset.

The two new mutations were found in girls with severe encephalopathy, autistic characteristics, severe deterioration of psychomotor development and onset of seizures within the first month of life, although there were some phenotypic differences between the two cases. These characteristics are in agreement with Bahi-Buisson *et al.*[31] and other authors [6,32], who linked mutations in the *CDKL5* gene with various and overlapping phenotypes, ranging from autism and mental retardation to RTT with epilepsy phenotypes.

Recent studies have focused on better characterising the relationships between genotypes and phenotypes, given that phenotypic heterogeneity caused by mutations in *CDKL5* is attributable to the nature of the mutations and their location, to inactivation of the X chromosome, and to cellular differences caused by these various mutations [31]. Some authors have failed to find any clear relationship between the type of mutation and the severity of the phenotype [31], but the most recent studies suggest that mutations in the catalytic domain of the protein (from exon 2 to exon 12; the pathogenic mutations in the present study were identified in exons 8 and 10) cause more severe clinical characteristics [7,26,32]. Specifically, Cástren *et al.*[33] reported that an onset of epileptic seizures within the first 6 months of life distinguished patients with *CDKL5* mutations from patients with typical RTT caused by *MECP2* mutations, which is in accordance with the results of the current study.

There is evidence that CDKL5 interacts with MeCP2 and modifies its phosphorylation. Castrén *et al.*[33] suggested that *MECP2* expression levels regulate the methylation-dependent binding of CDKL5 to MeCP2 and that this interaction may explain the finding that *CDKL5* mutations produce a phenotype with some features that overlap with RTT. Other authors [31,34] have speculated that the variable clinical presentations of *CDKL5*-related encephalopathy result from the transcriptional or translational effects of *CDKL5* mutations. Further studies are required to clarify the physiological interactions between these genes.

X-chromosome inactivation is known to impact on the clinical manifestations of X-linked disorders; however, this point cannot be investigated in studies that use blood, rather than brain cells [8,31], where preferential inactivation of the mutated chromosome is believed to occur [33]. Specifically, patients with pathological mutations in the current study had normal, random inactivation of the X chromosome (58:42 and 56:44).

Finally, this study highlights the importance of analysing rearrangements of the *CDKL5* gene; according to Mei *et al.*[35], more than 10% of the mutations in the *CDKL5* gene are rearrangements. Two new pathogenic mutations were identified in the present cohort, one of which was due to a deletion detected using MLPA (50%).

## Conclusions

The results of this study confirm that *CDKL5* mutations are a potentially important etiological factor in neurodevelopmental disorders. Females with RTT-like phenotypes, severe encephalopathy, and very early-onset epilepsy may benefit from a complete analysis of the *CDKL5* gene, in terms of both its sequence and its rearrangements. Early onset was considered to be onset before the age of 5 months [1,36], which was the most consistent clinical sign associated with *CDKL5* mutations. In addition, the results demonstrate the usefulness of MLPA as a clinical screening method to establish a causative diagnosis in these patients. Overall, this study provides an important contribution to improving the understanding of the *CDKL5* gene and its pathology.

## Competing interests

The authors declare no conflict of interest.

## Authors’ contributions

MTC, RD, FR, AGR, MJM and MALA acquired and provided clinical data and samples from their patients. HM, CMB, NP and IR produced and analysed the molecular data. HM wrote the manuscript. MIT designed, supervised and directed the project and revised the manuscript. All authors read and approved the final manuscript.

## Pre-publication history

The pre-publication history for this paper can be accessed here:

http://www.biomedcentral.com/1471-2350/13/68/prepub

## References

[B1] Neul JL Kaufmann WE Glaze DG Christodoulou J Clarke AJ Bahi-Buisson N Leonard H Bailey ME Schanen NC Zappella M Renieri A Huppke P Percy AK RettSearch Consortium Rett syndrome: revised diagnostic criteria and nomenclature Ann Neurol 2010 68 6 944 950 10.1002/ana.22124 21154482 PMC3058521

[B2] Chacrour M Zoghby HY The story of Rett syndrome: from clinic to neurobiology Neuron 2007 56 422 437 10.1016/j.neuron.2007.10.001 17988628

[B3] Chen Q Zhu YC Yu J Miao S Zheng J Xu L Zhou Y Li D Zhang C Tao J Xiong ZQ CDKL5, a protein associated with Rett Syndrome, regulates neuronal morphogenesis via Rac1 signaling J Neurosci 2010 30 38 12777 12786 10.1523/JNEUROSCI.1102-10.2010 20861382 PMC6633570

[B4] Bahi-Busson N Kaminska A Boddaert N Rio M Afenjar A Gérard M Giuliano F Motte J Héron D Morel MA Plouin P Richelme C des Portes V Dulac O Philippe C Chiron C Nabbout R Bienvenu T The three stages of epilepsy in patients with CDKL5 mutations Epilepsia 2008 49 6 1027 1037 10.1111/j.1528-1167.2007.01520.x 18266744

[B5] Sprovieri T Conforti FL Fiumara A Mazzei R Húngaro C Citrigno L Muglia M Arena A Quattrone A A novel mutation in the X-linked cyclin-dependent kinase-like 5 (CDKL5) gene associated with a severe Rett phenotype Am J Med Genet A 2009 149A 4 722 725 10.1002/ajmg.a.32711 19253388

[B6] Mari F Azimonti S Bertani I Bolognese F Colombo E Caselli R Scala E Longo I Grosso S Pescucci C Ariani F Hayek G Balestri P Bergo A Badaracco G Zappella M Broccoli V Renieri A Kilstrup-Nielsen C Landsberger N CDKL5 belongs to the same molecular pathway of MECP2 and it is responsible for the early-onset seizure variant of Rett syndrome Hum Mol Genet 2005 14 14 1935 1946 10.1093/hmg/ddi198 15917271

[B7] Russo S Marchi M Cogliati F Bonati MT Pintaudi M Veneselli E Saletti V Balestrini M Ben-Zeev B Larizza L Novel mutations in the CDKL5 gene, predicted effects and associated phenotypes Neurogenetics 2009 10 3 241 250 10.1007/s10048-009-0177-1 19241098

[B8] Nemos C Lambert L Giuliano F Doray B Roubertie A Goldenberg A Delobel B Layet V Nguyen MA Saunier A Verneau F Jonveaux P Philippe C Mutational spectrum of CDKL5 in early-onset encephalopathies: a study of a large collection of French patients and review of the literature Clin Genet 2009 76 357 371 10.1111/j.1399-0004.2009.01194.x 19793311

[B9] Rademacher N Hambrock M Fischer U Moser B Ceulemans B Lieb W Boor R Stefanova I Gillessen-Kaesbach G Runge C Korenke GC Spranger S Laccone F Tzschach A Kalscheuer VM Identification of a novel CDKL5 exon and pathogenic mutations in patients with severe mental retardation, early-onset seizures and Rett-like features Neurogenetics 2011 12 2 165 167 10.1007/s10048-011-0277-6 21318334

[B10] Scala E Ariani F Mari F Caselli R Pescucci C Longo I Meloni I Giachino D Bruttini M Hayek G Zappella M Renieri A CDKL5/STK9 is mutated in Rett syndrome variant with infantile spasms J Med Genet 2005 42 2 103 107 10.1136/jmg.2004.026237 15689447 PMC1735977

[B11] Kalscheuer VM Tao J Donnelly A Hollway G Schwinger E Kübart S Menzel C Hoeltzenbein M Tommerup N Eyre H Harbord M Haan E Sutherland GR Ropers HH Gécz J Disruption of the serine/threonine kinase 9 gene causes severe X-linked infantile spasms and mental retardation Am J Hum Genet 2003 72 6 1401 1411 10.1086/375538 12736870 PMC1180301

[B12] Evans JC Archer HL Colley JP Ravn K Nielsen JB Kerr A Williams E Christodoulou J Gécz J Jardine PE Wright MJ Pilz DT Lazarou L Cooper DN Sampson JR Butler R Whatley SD Clarke AJ Early onset seizure and Rett-like features associated with mutations in CDKL5 Eur J Hum Genet 2005 13 10 1113 1120 10.1038/sj.ejhg.5201451 16015284

[B13] Beristain E Martínez-Bouzas C Guerra I Viguera N Moreno J Ibañez E Díez J Rodríguez F Mallabiabarrena G Luján S Gorostiaga J De Pablo JL Mendizábal JL Tejada MI Differences in the frequency and distribution of BRCA1 and BRCA2 mutations in breast/ovarian cancer cases from the Basque country with respect to the Spanish population: implications for genetic counselling Breast Cancer Res Treat 2007 106 255 262 10.1007/s10549-006-9489-0 17262179

[B14] Bijlsma EK Collins A Papa FT Tejada MI Wheeler P Peeters EA Gijsbers AC van de Kamp JM Kriek M Losekoot M Broekma AJ Crolla JA Pollazzon M Mucciolo M Katzaki E Disciglio V Ferreri MI Marozza A Mencarelli MA Castagnini C Dosa L Ariani F Mari F Canitano R Hayek G Botella MP Gener B Mínguez M Renieri A Ruivenkamp CA Xq28 duplications including MECP2 in five females: Expanding the phenotype to severe mental retardation Eur J Med Genet 2012 55 6-7 404 413 Mar 29. [Epub ahead of print] 10.1016/j.ejmg.2012.02.009 22522176 PMC3383992

[B15] International Rett Syndrome Foundation (IRSF) [http://mecp2.chw.edu.au/cgi-bin/mecp2/views/basic.cgi?form=basic]

[B16] BIOBASE-HGMD [https://portal.biobase-international.com/hgmd/pro/gene.php?gene=cdkl5]

[B17] HGVS [http://grenada.lumc.nl/LOVD2/MR/home.php?select_db=CDKL5]

[B18] NCBI, SNPs: dbSNPs [http://www.ncbi.nlm.nih.gov/SNP/snp_ref.cgi?locusId=6792]

[B19] Database of Genomic Variants [http://projects.tcag.ca/cgi-bin/variation/tbrowse?source=hg18&table=Locus&show=table&keyword=&rnum=0&flop=AND&fcol=_C2&fcomp=in&fkwd=Variation_83380,Variation_83381,Variation_83382,Variation_96514&cols=]

[B20] DECIPHER [https://decipher.sanger.ac.uk/search?q=CDKL5]

[B21] UCSC [http://genome.ucsc.edu/cgi-bin/hgTracks?db=hg19&omimGene=full&decipher=full&position=ChrX:17842369–18552860]

[B22] ENSEMBLE [http://www.ensembl.org/Homo_sapiens/Search/Results?species=Homo_sapiens;idx=;q=cdkl5]

[B23] Genecard [http://www.genecards.org/cgi-bin/carddisp.pl?gene=CDKL5&search=CDKL5]

[B24] 1000 Genomes [http://browser.1000genomes.org/Homo_sapiens/Gene/Variation_Gene/Table?db=core;g=ENSG00000008086;r=X:18443703–18671749]

[B25] ESE Finder program [http://rulai.cshl.edu/cgi-bin/tools/ESE3/esefinder.cgi?process=home]

[B26] Nectoux J Heron D Tallot M Chelly J Bienvenu T Maternal origin of a novel C-terminal truncation mutation in CDKL5 causing a severe atypical form of Rett syndrome Clin Genet 2006 70 1 29 33 10.1111/j.1399-0004.2006.00629.x 16813600

[B27] Intusoma U Hayeeduereh F Plong-On O Sripo T Vasiknanonte P Janjindamai S Lusawat A Thammongkol S Visudtibhan A Limprasert P Mutation screening of the CDKL5 gene in cryptogenic infantile intractable epilepsy and review of clinical sensitivity Eur J Paediatr Neurol 2011 15 5 432 438 10.1016/j.ejpn.2011.01.005 21775177

[B28] Tao J Van Esch H Hagedorn-Greiwe M Hoffmann K Moser B Raynaud M Sperner J Fryns JP Schwinger E Gécz J Ropers HH Kalscheuer VM Mutations in the X-linked Cyclin-Dependent Kinase-like 5 (CDKL5/STK9) gene are associated with severe neurodevelopmental retardation Am J Hum Genet 2004 75 1149 1154 10.1086/426460 15499549 PMC1182152

[B29] Stalpers XL Spruijt L Yntema HG Verrips A Clinical phenotype of 5 females with a CDKL5 mutation J Child Neurol 2012 27 1 90 3 10.1177/0883073811413832 21765152

[B30] Saitsu H Osaka H Nishiyama K Tsurusaki Y Doi H Miyake N Matsumoto N A girl with early-onset epileptic encephalopathy associated with microdeletion involving CDKL5 Brain Dev 2012 34 5 364 367 Jul 27. [Epub ahead of print] 10.1016/j.braindev.2011.07.004 21802232

[B31] Bahi-Buisson N Nectoux J Rosas-Vargas H Milh M Boddaert N Girard B Cances C Ville D Afenjar A Rio M Héron D Nguyen Morel MA Arzimanoglou A Philippe C Jonveaux P Chelly J Bienvenu T Key clinical features to identify girls with CDKL5 mutations Brain 2008 131 2647 2661 10.1093/brain/awn197 18790821

[B32] Psoni S Willems PJ Kanavakis E Mavrou A Frissyra H Traeger-Synodinos J Sofokleous C Makrythanassis P Kitsiou-Tzeli S A novel p.Arg970X mutation in the last exon of the CDKL5 gene resulting in late-onset seizure disorder Eur J Paedriatr Neurol 2010 14 2 188 191 10.1016/j.ejpn.2009.03.006 19428276

[B33] Castrén M Gaily E Tengström C Lähdetie J Archer H Ala-Mello S Epilepsy caused by CDKL5 mutations Eur J Paediatr Neurol 2011 15 1 65 69 10.1016/j.ejpn.2010.04.005 20493745

[B34] Mastrangelo M Vincenzo L Genes of early-onset epileptic encephalopathies: from genotype to phenotype Pediatr Neurol 2012 46 1 24 31 10.1016/j.pediatrneurol.2011.11.003 22196487

[B35] Mei D Marini C Novara F Bernardina BD Granata T Fontana E Parrini E Ferrari AR Murgia A Zuffardi O Guerrini R Xp22.3 genomic deletions involving the CDKL5 gene in girls with early onset epileptic encephalopathy Epilepsia 2010 51 4 647 654 10.1111/j.1528-1167.2009.02308.x 19780792

[B36] Artuso R Mencarelli MA Polli R Sartori S Ariani F Pollazzon M Marozza A Cilio MR Specchio N Vigevano F Vecchi M Boniver C Dalla Bernardina B Parmeggiani A Buoni S Hayek G Mari F Renieri A Murgia A Early-onset seizure variant of Rett syndrome: definition of the clinical diagnostic criteria Brain Dev 2010 32 17 24 10.1016/j.braindev.2009.02.004 19362436

